# Improving physical function with physiotherapy assistants following intensive care unit admission (EMPRESS): A randomised controlled feasibility study

**DOI:** 10.1177/17511437251328899

**Published:** 2025-05-16

**Authors:** Rebecca J Cusack, Andrew Bates, Hannah Golding, Kay Mitchell, Linda Denehy, Nicholas Hart, Ahilanandan Dushianthan, Gordon Sturmey, Iain Davey, Zoe van Willigen, Sarah Elliott, Laura Ortiz-RuizDeGordoa, Jessica Cooper, Barbara Philips, Jenny Rains, Sally Pitts, Nigel Beauchamp, Isabel Reading, Mike Grocott

**Affiliations:** 1 University of Southampton, Southampton, UK; 2 NIHR Southampton Biomedical Research Centre, University Hospital Southampton NHS Foundation Trust, University of Southampton, Southampton, UK; 3 University of Melbourne, Melbourne, Australia; 4 Peter MacCallum Cancer Centre, Melbourne, VIC, Australia; 5 Lane Fox Clinical Respiratory Physiology Research Centre, Guys and St Thomas’ Hospital NHS Foundation Trust, London, UK; 6 Centre for Human and Applied Physiological Science, King’s College London, UK; 7 Independent ICU Patient Representative, Thatcham, UK; 8 Independent ICU Patient Representative, Poole, UK; 9 North Cumbria Integrated Care NHS Foundation trust, Carlisle, UK; 10 Brighton and Sussex University Hospitals NHS Foundation Trust, UK; 11 Royal Bournemouth Hospital University Hospitals Dorset NHS Foundation Trust, UK

**Keywords:** Feasibility, critical care, humans, exercise therapy, physical therapy modalities, physiotherapy assistant, protocolised rehabilitation, cycle ergometry

## Abstract

**Introduction:**

Early rehabilitation of critically ill patients is challenging due to limited staff resources. This study assessed the feasibility of delivering a randomised controlled trial of physiotherapy assistants delivering early protocolised rehabilitation plus usual care compared with usual care.

**Methods:**

We conducted a randomised feasibility study in three U.K. mixed medical/surgical intensive care units. Eligible patients were intubated and ventilated <72 h, expected to be ventilated for a further 48 h, and functionally independent before ICU admission. Patients were randomised to protocolised early rehabilitation plus usual care or usual care. Feasibility outcomes were (i) recruitment of one to two patients/per month/site; (ii) >75% of patients commencing the intervention within 72 h of ventilation with >70% interventions delivered; and (iii) blinded outcome measures recorded at three-time points in >80% of patients.

**Results:**

The study delivery was compromised by the COVID-19 pandemic: 46 patients were enrolled, of which 22 were allocated to intervention. Feasibility outcomes: (i) recruitment of 0.9 patients/month/site, (ii) 90% of patients commenced interventions within 72 h of ventilation, with 166/264 (63%) of study interventions delivered: median total 22.5 min (IQR 15–35) of therapy per day in the usual care group and 45 min (IQR 25–70) in the intervention group, and (iii) the outcome assessments were performed at three-time points for 64% of survivors, 63% of which were blinded.

**Conclusion:**

While delivery of protocolised rehabilitation by physiotherapy assistants is feasible, the design of a future RCT needs to consider strategies to improve recruitment and complete blinded outcome assessments.

## Background

Early rehabilitation interventions in critically ill patients may reduce the duration of mechanical ventilation, Intensive Care Unit (ICU) and hospital length of stay (LOS)^1^ and improve physical,^2,3^ cognitive^4^ and psychiatric well-being.^5^ However, uptake is variable, and reported benefits are inconsistent and often fail to translate into improved long-term outcomes.^1^ Moreover, recent safety concerns have further increased scrutiny regarding what interventions are delivered and when.^3,6^

Despite this inconclusive evidence, eight international guidelines recommend early ICU mobilisation and rehabilitation,^7^ although clinical implementation remains challenging. Identified barriers include lack of staff and resources, patient factors, notably heavy sedation and clinical instability, and lack of clinician buy-in.^8,9^

With the potential for both clinical,^1–5^ and economic benefits^10,11^ of early rehabilitation, we previously introduced an early rehabilitation pathway using physiotherapy assistants to work alongside senior physiotherapy staff within our teaching hospital ICU.^12^ This approach enabled the delivery of more therapy interventions while freeing up senior physiotherapy staff. This current study aimed to investigate the feasibility of introducing protocolised early rehabilitation delivered by physiotherapy assistants and compare it to usual care in mechanically ventilated ICU patients. The results will inform the design of a larger RCT.

## Methods

### Study design and setting

This study was designed as a two-centre feasibility study using a two-arm RCT with 1:1 randomisation and blinded outcome assessments completed at ICU discharge, hospital discharge and 3-month follow-up. The entire protocol is published elsewhere.^13^ With the approval of the trial steering committee, a third centre joined the study in January 2022 due to recruitment challenges exacerbated by the prolonged reallocation of research staff to clinical delivery during the SARS-CoV-2 pandemic in 2020/2021. Ethical approval was granted by the UK South-Central Hampshire (A) Research Ethics Committee (19/SC/0016) and registered on ClinicalTrials.gov (NCT03771014).

The staffing models, usual physiotherapy practices and study delivery teams varied by site, reflecting each unit’s configuration (Supplemental Table 1a and 1b). The two original sites received study funding for a full-time physiotherapy assistant. Funding constraints did not allow the employment of an additional physiotherapy assistant at the third site to facilitate the delivery of the study. However, this site had higher staff ratios, which enabled the study to be delivered by a junior physiotherapist without extra costs. The junior physiotherapist assisted in the additional rehabilitation sessions and provided support comparable to the physiotherapy assistants at the other sites.

### Participants

In each centre, an ICU research coordinator screened all mechanically ventilated ICU patients to identify those who met the study inclusion criteria. To reduce cohort heterogeneity and target patients with the greatest potential to benefit from the rehabilitation interventions, patients were recruited within 72 h of initiating intubation and mechanical ventilation, were >42 years old,^14^ previously functionally independent, as indicated by a Barthel score > 80 and admitted as an unplanned medical admission to the ICU. Exclusion criteria were being a hospital inpatient for 5 days or more before ICU admission, acute brain or spinal cord injury, known or suspected neurological/muscular impairment, a condition limiting use of cycle ergometry (e.g. lower limb fracture/amputation), considered unlikely to survive >48 h by the consultant intensivist in charge of the patient’s care, persistent therapy exemptions in the first 3 days of mechanical ventilation and patients recruited to another study without a co-enrolment agreement in place. Written informed consent was obtained before randomisation. The initial agreement for enrolment was obtained from a personal consultee of the patient or, if unavailable, a professional consultee. Subsequent patient consent was obtained on recovery of capacity.

### Randomisation

Participants were registered on a bespoke electronic data collection tool (ALEA Clinical B.V) and randomly assigned (1:1) to the protocolised early rehabilitation or usual care by a person not involved in intervention or outcome assessment. Data was collected concurrently and entered directly into the electronic database by research team members.

### Interventions

The attending clinician and senior research physiotherapist jointly determined the patient’s suitability to be enrolled in the study. Due to the nature of the intervention, it was not possible to blind staff or participants to randomisation allocations. Patients were assigned to receive usual care or usual care plus the study rehabilitation intervention, this being two 30-min protocolised rehabilitation sessions 5 days/week (details previously reported).^13^ The intervention sessions commenced as soon as practically possible after randomisation within the described safety criteria (Supplemental Table 2). Where feasible, usual physiotherapy care sessions in both arms of the study were provided by the usual physiotherapy team, who were not involved in the study. The study physiotherapy assistant and senior physiotherapist in the research team delivered the early rehabilitation intervention sessions. The research physiotherapist evaluated the patient before each rehabilitation intervention. At the two sites with a physiotherapy assistant, in-bed interventions were delivered by the physiotherapy assistant in the presence of the bedside nurse, with the supervising physiotherapist remaining in proximity. At the third site, the junior physiotherapist, separate from the usual care physiotherapy team, took on the role of the physiotherapy assistant. Higher levels of mobility, from sitting on the edge of the bed to walking, were delivered by the senior research physiotherapist, the physiotherapy assistant and the bedside nurse. Sedation was assessed and recorded at the start of each physiotherapy session using the Richmond Agitation–Sedation Scale (RASS)^15,16^ with a target sedation level between −1 and +1.

The first intervention session each day included a passive or active range of movements, passive cycling, active cycling, in-bed exercises, sitting, mobilisation out of bed and walking. The mode of intervention was chosen at the discretion of the overseeing physiotherapist to achieve the highest possible level of activity, given safety considerations and the patient’s capability. The second intervention session aimed to deliver a 30-min protocolised cycling using an in-bed supine cycle ergometer (MotoMed Letto 2 Reck-Technik GmbH, Germany). As previously detailed, the cycling protocol consisted of a 5-min warm-up, followed by up to 20 min of passive or active cycling, dependent on ability, finishing with a 5-min cool-down.^13^ If patients were considered unable to have concurrent rehabilitation interventions and respiratory weaning, rehabilitation interventions took priority, in agreement with the senior clinical team. All patients received usual physiotherapy interventions from ICU admission through to discharge. Participants in the rehabilitation arm received additional interventions that continued for 28 days or until ICU discharge.

### Safety considerations

Patients were monitored for cardiovascular and respiratory stability and safety of indwelling lines, tubes and catheters with predetermined criteria for termination of any session. Adverse events were managed according to standard operating procedures within the ICUs. Concerns by the research and bedside nursing staff were escalated to the medical team. Any events were documented and reported to the safety monitoring committee. All adverse safety events were defined as any intervention ceased according to stopping criteria (Supplemental Table 2).

### Feasibility outcomes

The predefined primary objective was to assess the feasibility of delivering the designed protocol to inform a future RCT. Feasibility outcomes were: (i) recruitment of one to two patients per month per site; (ii) protocol fidelity with >75% of patients commencing interventions within 72 h of mechanical ventilation, with >70% interventions delivered; and (iii) blinded outcome measures recorded by physiotherapists, at three time points in >80% of survivors.

### Secondary outcomes

The secondary outcome measures planned for a definitive trial are in Supplemental Table 3. Study participants and their families/next of kin were asked not to reveal their treatment allocation to the blinded assessors.

### Sample size and statistical analysis

No formal sample size calculation was undertaken for this feasibility study. We aimed to recruit 90 patients, 30–45 participants at each site. We anticipated a 30% in-hospital mortality/loss to follow-up, with an estimate of 60 patients completing the study.

Feasibility outcomes (recruitment, adherence, and retention rates) are presented descriptively across the study population. Clinical outcome data (secondary outcomes) are presented as summary statistics using means (SDs) or medians (ranges/IQRs) as applicable across the whole study population and by treatment arm. Data were entered directly into a bespoke secure electronic case report form (ALEA). Data validation occurred according to the procedures set out in the data management and validation plans; both developed a priori. Data were directly extracted from the electronic database and analysed using STATA/SE 16.1 statistical software (StataCorp, USA, 2019).

## Results

**Figure 1.**
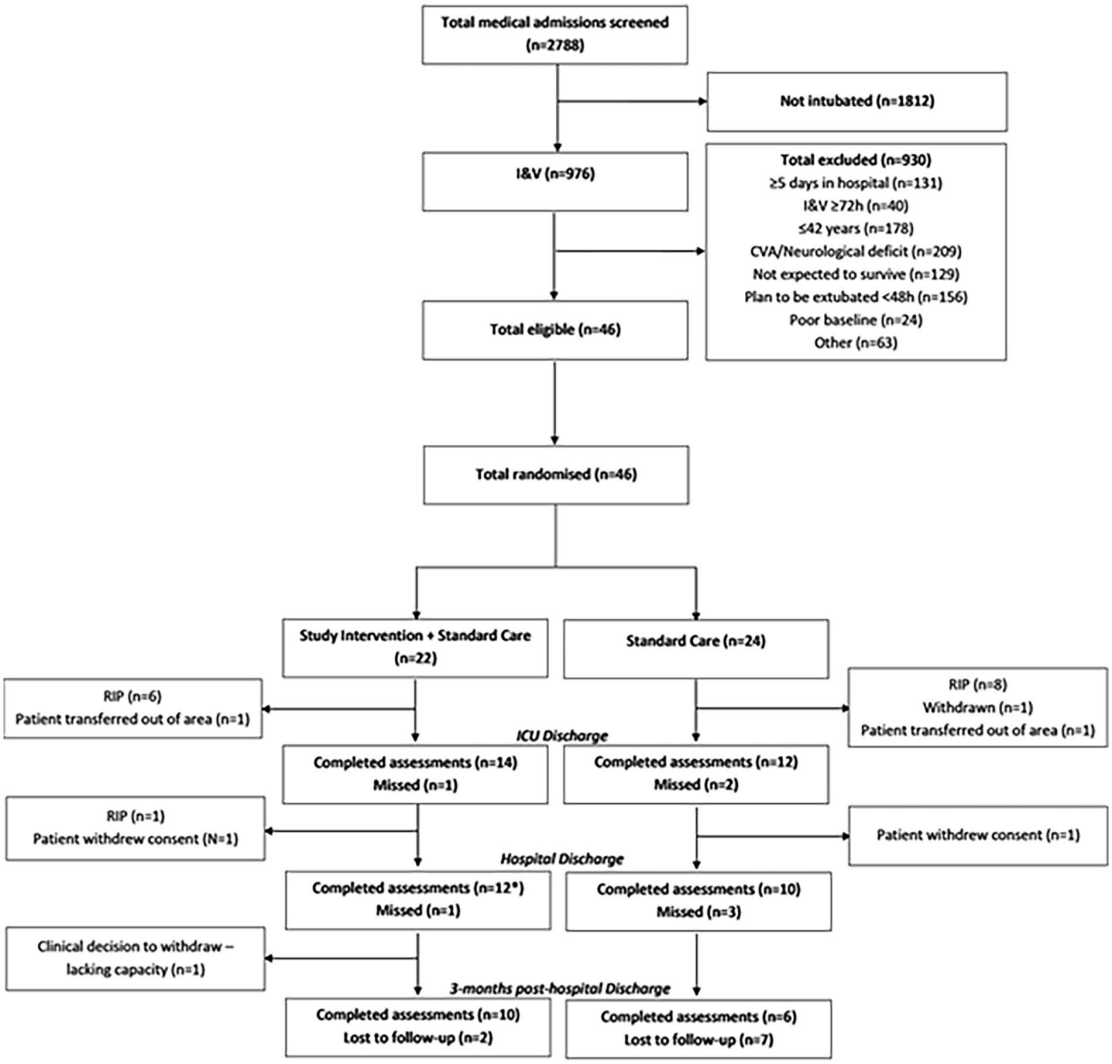
Patient flow diagram. *n = 2 same assessment as ICU discharge (ICU to home).

Resource limitations and hospital restrictions during the COVID-19 pandemic significantly compromised the study delivery. Details of participant flow and characteristics are outlined in Figure 1 and Table 1. Feasibility outcomes are detailed in Table 2.

**Table 1.** Patient characteristics.

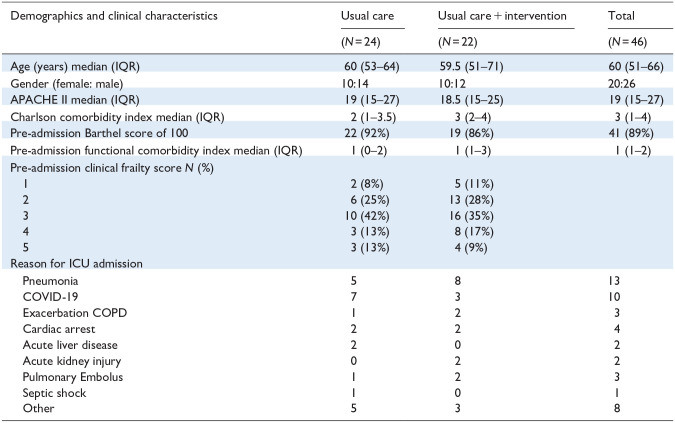

**Table 2.** Feasibility outcomes.

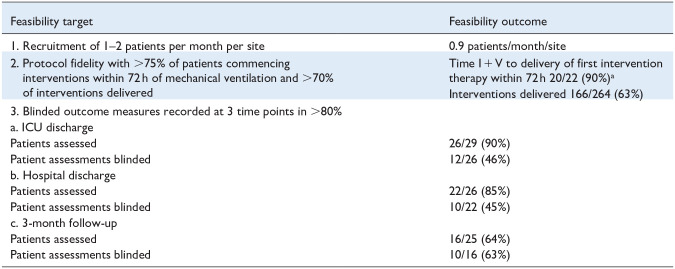

a 2 patients in the intervention plus usual care arm did not receive any study rehabilitation interventions. One was due to clinical instability, and the other was due to recruitment on Friday and a lack of research staff over the weekend. All 20 patients who received intervention physiotherapy did so within 72 h of I + V.

Feasibility outcomes are detailed in Table 2.

### Recruitment

Forty-six patients were recruited between June 2019 and June 2022, with the study suspended between 12th March 2020 and 31st July 2020 due to restrictions imposed due to the SARS-CoV2 pandemic. The reallocation of research staff to clinical delivery resulted in continued reduced research activity throughout 2020/2021, with one of the sites not able to recruit patients for 8 months. The third site opened for recruitment in January 2022. The study was discontinued before the recruitment goal was achieved due to below-target recruitment and exhaustion of funding. Consultee assent was obtained for all participants who were approached. However, two participants later withdrew from the study based on clinician decision due to clinical instability. Additionally, two participants withdrew: one did not wish to return to the hospital for follow-up assessments, and the second did not wish to have further data collected. Sites were not recruiting simultaneously. The total sum of months each site was open to recruitment was 51 months. The duration of recruitment and accruals of patients per month at each site was 19 months (0.6 pt/month), 23 months (1.2 pt/month) and 9 months (0.8 pt/month) at sites 1, 2 and 3, respectively. Overall recruitment across the sites was 0.9 pt/month. Interventions delivered are detailed in Table 3.

**Table 3.** Interventions.

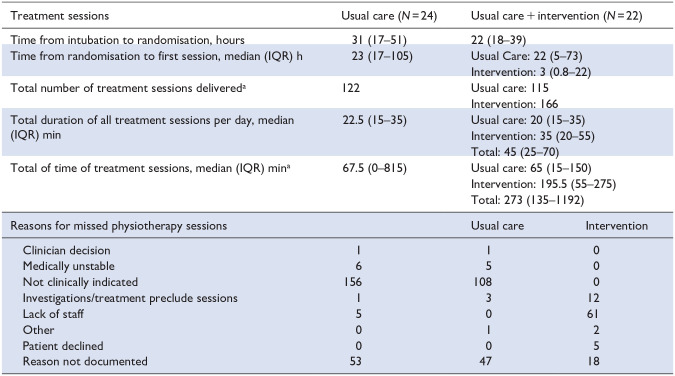

It is usual practice for physiotherapy staff to have dedicated sessions within ICUs in the UK.

a Seven patients in the usual care arm and 2 in the intervention arm received no treatment sessions.

### Protocol fidelity

In the intervention arm, 20/22 (90%) patients received their first rehabilitation session within 72 h of mechanical intubation. The two remaining patients did not receive any rehabilitation interventions due to clinical instability. The median time from intubation to the first intervention was 37 h (IQR 19–59). Of the two potential early rehabilitation interventions per weekday, 63% (166/264) were delivered. The median total physical therapy intervention time was 67.5 min (IQR 0–815) in the usual care group compared to 273 min (IQR 135–1192) in the intervention group. On weekdays, when dedicated physiotherapy service is available, the usual care group received a median of 21.5 min (IQR 15–35) of physiotherapy sessions compared to the intervention group, which received 45 min (IQR 25–70). 166 /264 (63%) of planned rehabilitation intervention sessions were delivered. The primary reason for the non-delivery of planned rehabilitation intervention therapy sessions was a lack of staff availability, accounting for 61/98 (62%) missed sessions (Table 3). The intervention group received a median additional 35 min (IQR 20–55) of the target of 60 min of early rehabilitation therapy per day.

RASS scores were recorded for the initial physiotherapy session for 21 patients and were below target RASS across both study groups, with all scores ranging between −5 and 3, with over half (52%) scoring −5.

### Blinded outcome assessments

Study participants and their families/next of kin were asked not to reveal their treatment allocation to the blinded assessors. The overall mortality was 15/46 (33%) at 3 months. Survivors’ ICU and hospital discharge outcome assessments were completed in 90% and 85% of surviving participants, respectively. The proportion of these assessments that were blinded was below target at all three-time points. Outcome assessments were conducted at three time points in 16/25 patients alive at 3 months, and 10 of these 16 assessments were blinded. Only 50% of assessments completed were delivered by a blinded assessor. Secondary physical outcome assessments at ICU discharge are shown in Supplemental Table 4. The 3-month assessments, as detailed in Table 4, were notably impacted by COVID-19 restrictions on hospital attendance.

**Table 4.** Patient outcomes.

Secondary Outcomes	Usual care	Usual care + intervention	Total
	(*N* = 24)	(*N* = 22)	(*N* = 46)
Duration of ventilation (days)^a^	7 (4–10.5)	7 (3–10)	7 (4–10)
ICU length of stay (days)^a^	10 (6–21)	11 (7–16)	10 (7–17.5)
ICU mortality	8 (33%)	6 (27%)	14 (30%)
Hospital length of stay (days)^a^	16.5 (9–33)	15 (10.5–39)	16 (9–33)
Hospital mortality	8 (33%)	7 (32%)	15 (32%)
Alive at 3-months	16 (67%)	15(68%)	31 (67%)
Available data at 3 months	*N* = 6	*N* = 10	*N* = 16
HADS total^a^	10.5 (4–13)	7 (4–13)	9 (4–13)
HADS anxiety^a^	5.5 (2–10)	3 (2–7)	4 (2–7.5)
HADS depression^a^	4 (2–5)	3.5 (2–9)	3.5 (2–8)
IES-R^a^	26 (7–35)	19.5 (12–26)	20.5 (9.5–29)
WHODAS2^a^	21 (18–27)	7(4–30)	
EQ-5D-5L^a^	12.5(9–15)	7.5(7–12)	

HADS: Hospital Anxiety and Depression Score; IES-R: Impact Event Scale-Revised; WHODAS 2: World Health Organization Disability Assessment Schedule 2.0; EQ-5D-5L: EuroQol-5 Dimensions-5 Levels.

a Median (IQR).

### Adverse events

No serious adverse events occurred. Two adverse events were reported during 433 therapy sessions delivered (0.69% per session). One patient in the intervention arm had a 30-s, self-terminating episode of ventricular tachycardia during a cycle intervention without lasting consequences. This intervention session was immediately terminated. One patient in the intervention arm suffered minor skin abrasions from the cycle strapping, noticed after the therapy session had finished. Neither of the adverse events required medical intervention.

## Discussion

This study evaluated the feasibility of conducting a randomised controlled trial involving physiotherapy assistants delivering early protocolised rehabilitation. Despite the challenges posed by the SARS-CoV-2 pandemic, which led to only partial success, the study provided valuable insights for the design of a larger RCT. The intervention was initiated well within the target window of 72 h, and although the overall duration of the intervention was high, the number of interventions and patient accrual fell slightly short of the target. The desired levels of blinded outcome assessments were not achieved.

This is the first study to successfully deliver a protocolised early rehabilitation pathway using therapy assistants supported by physiotherapy staff. We came close to achieving our target monthly recruitment rate of 1–2 patients. Other studies with broader inclusion time windows,^17–19^ recruiting both medical and surgical patients^18,20,21^ and patients receiving both invasive and non-invasive ventilation^20^ also report recruitment challenges.

Our study focussed on a narrow cohort, excluding patients under 42 years of age, to ensure those more likely to benefit from early rehabilitation were included.^14,22^ This criterion accounted for 19% of excluded patients. An additional 22.5% were excluded due to suspected or confirmed permanent neurological deficits, and 40 eligible patients were not recruited within the 72-h window due to limited weekend staffing and staff reallocations. Despite our strict inclusion criteria and pandemic-related recruitment challenges, our patient accruals averaged close to one per month, consistent with similar studies.^19^

Different patient cohorts will likely benefit from different rehabilitation strategies.^23^ The optimal timing to initiate rehabilitation interventions, along with frequency, duration, mode and intensity, remains unclear^23–27^; however, evidence suggests initiation between 24 and 72 h may be the most beneficial.^28^ We excluded patients who had been hospitalised for more than 5 days before ICU admission to avoid including those who may have established deconditioning. Additionally, we enrolled patients within 72 h of ICU admission due to the well-documented rapid and early muscle breakdown that occurs at the onset of critical illness.^29–31^

Physiotherapy services vary across healthcare settings,^32–34^ and the availability of dedicated physiotherapy staff is a significant barrier to delivering early rehabilitation interventions.^8,17,20,35–38^ A novel aspect of our study was employing physiotherapy assistants supervised by physiotherapists to provide early rehabilitation. With this staff structure, not only were early rehabilitation interventions commenced within a median of 3 h (IQR 0.8–22) of randomisation, but the median total time of therapy treatment sessions delivered per day doubled. We have demonstrated that supervised physiotherapy assistants can safely and effectively facilitate the implementation of early rehabilitation. While it is acknowledged that having a junior physiotherapist deliver the interventions at the third site could offer potential benefits, the impact is difficult to characterise. The interventions that could be delivered by and under the supervision of the physiotherapy assistants are described in the protocol. Usual care provided by physiotherapists not involved in delivering the rehabilitation interventions was comparable in duration between the groups. Although only 63% of potential intervention sessions were delivered, patients in the intervention group received a median of 45 min of total therapy interventions per day, compared to a median of 22.5 min in the usual care group (Table 3). The intervention targeted delivering two additional 30-min therapy sessions per day. However, the first session aimed at achieving the highest possible level of activity for the patient, with the session ending once this level of activity was achieved. This level of mobility was often reached before 30 min, resulting in a total intervention time of less than 60 min, even when both additional therapy sessions were delivered. Even with physiotherapy assistants, the most common reason for missed intervention sessions was a lack of staff availability. This may, in part, be attributed to staff relocation during the pandemic; additionally, with only one extra staff member, there was no cover for sickness or annual leave. This contrasts with other RCTs, which report limited cohort separation with up to only 13 min of additional physiotherapy intervention time.^6,17,19,20^ Notably, a recent observational study reported that 40 min of physiotherapy intervention improves patient outcomes at ICU discharge.^39^

We achieved high overall assessment rates, although conducting the gold standard of blinded outcome assessments proved challenging in our study, as it has for other investigators.^1^ This difficulty was due to the small size of the research teams at two of the three sites, which limited the availability of blinded assessors. A number of strategies could help address these challenges in future trials. These include establishing a dedicated team of blinded assessors independent from the intervention team and carefully planning when, where, and how outcomes are assessed. Additionally, outcome measures for a future trial should align with the recently published core outcome set recommendations for critical care rehabilitation trials.^40^

Even with the challenges, our study provides several insights to inform the development of a subsequent RCT. We enrolled a well-defined and homogenous cohort, focussing on patients most likely to benefit from early physiotherapy interventions. To ensure consistent delivery of the protocolised rehabilitation pathway across sites, we developed a bespoke education package and held biweekly troubleshooting meetings. These interactions facilitated protocol adherence and timely issue resolution. Our bespoke electronic data collection tool proved easy to use at the bedside and facilitated comprehensive data collection; however, contemporaneous data verification may have identified areas of difficulty with protocol delivery.

Key achievements include initiating rehabilitation interventions within 72 h of ICU admission and delivering a median of 35 min/day of these interventions in addition to usual care. This is challenging to achieve in routine ICU settings, and the support of dedicated physiotherapy assistants was crucial for delivering our protocol. Importantly, usual care interventions were comparable between groups, indicating that the rehabilitation protocol did not alter usual care practice. This likely reflects our study team structure, where usual physiotherapy teams delivered usual care in both arms while a separate research team solely delivered the rehabilitation interventions. Further, the staff resources differed in each of the three study sites, supporting the robustness and transferability of this model to deliver rehabilitation effectively.

It is essential that future research into early rehabilitation of critically ill patients carefully considers the results of the recently reported TEAM trial.^6^ This is the largest reported RCT of early rehabilitation in the ICU, which targeted delivery of the highest possible level of mobilisation for as long as possible. The authors reported excess adverse events in their intervention group without improved functional benefit.^6^ With the growing number of inconclusive studies, it may be time to step back and focus on developing a better understanding of the basic pathophysiology and underlying disease process. Metabolic and mitochondrial dysfunction are known to impact the progression and recovery from critical illness.^41,42^ Given the evidence that intense exercise can negatively affect mitochondrial function even in healthy individuals,^43^ caution may be warranted in pushing physiological reserves to the limit to enhance rehabilitation in the critically ill.

## Conclusion

Although the feasibility aims were not met in this study, the results suggest it is possible to effectively deliver a protocolised early rehabilitation programme using physiotherapy assistants. The protocol warrants further consideration to optimise recruitment, which outcome measures should be selected, when these data are collected, and by whom.

## Supplemental Material

sj-docx-1-inc-10.1177_17511437251328899 – Supplemental material for Improving physical function with physiotherapy assistants following intensive care unit admission (EMPRESS): A randomised controlled feasibility study Supplemental material, sj-docx-1-inc-10.1177_17511437251328899 for Improving physical function with physiotherapy assistants following intensive care unit admission (EMPRESS): A randomised controlled feasibility study by Rebecca J Cusack, Andrew Bates, Hannah Golding, Kay Mitchell, Linda Denehy, Nicholas Hart, Ahilanandan Dushianthan, Gordon Sturmey, Iain Davey, Zoe van Willigen, Sarah Elliott, Laura Ortiz-RuizDeGordoa, Jessica Cooper, Barbara Philips, Jenny Rains, Sally Pitts, Nigel Beauchamp, Isabel Reading and Mike Grocott in Journal of the Intensive Care Society

## References

[1] Waldauf P Jiroutková K Krajčová A , et al. Effects of rehabilitation interventions on clinical outcomes in critically ill patients: systematic review and meta-analysis of randomized controlled trials. Crit Care Med 2020; 48: 1055–1065. 32345834 10.1097/CCM.0000000000004382

[bibr2-17511437251328899] Watanabe S Hirasawa J Naito Y , et al. Association between the early mobilization of mechanically ventilated patients and independence in activities of daily living at hospital discharge. Sci Rep 2023; 13: 4265–20230314. 36918635 10.1038/s41598-023-31459-1 PMC10015081

[bibr3-17511437251328899] Paton M Chan S Tipping CJ , et al. The effect of mobilization at 6 months after critical illness - meta-analysis. NEJM Evid 2023; 2: 20221220. 10.1056/EVIDoa2200234 38320036

[bibr4-17511437251328899] Patel BK Wolfe KS Patel SB , et al. Effect of early mobilisation on long-term cognitive impairment in critical illness in the USA: a randomised controlled trial. Lancet Respir Med 2023; 11: 563–572. 36693400 10.1016/S2213-2600(22)00489-1 PMC10238598

[5] Watanabe S Liu K Nakamura K , et al. Association between early mobilization in the ICU and psychiatric symptoms after surviving a critical illness: a multi-center prospective cohort study. J Clin Med 2022; 11: 13. 10.3390/jcm11092587 PMC9099642 35566716

[6] Hodgson CL Bailey M Bellomo R , et al. Early active mobilization during mechanical ventilation in the ICU. N Engl J Med 2022; 387: 1747–1758. 36286256 10.1056/NEJMoa2209083

[7] Lang JK Paykel MS Haines KJ , et al. Clinical practice guidelines for early mobilization in the ICU: a systematic review. Crit Care Med 2020; 48: E1121–E1128. 10.1097/CCM.0000000000004574 32947470

[8] Dubb R Nydahl P Hermes C , et al. Barriers and strategies for early mobilization of patients in intensive care units. Ann Am Thorac Soc 2016; 13: 724–730. 27144796 10.1513/AnnalsATS.201509-586CME

[9] Parry SM Knight LD Connolly B , et al. Factors influencing physical activity and rehabilitation in survivors of critical illness: a systematic review of quantitative and qualitative studies. Intensive Care Med 2017; 43: 531–542. 28210771 10.1007/s00134-017-4685-4

[10] Murooka Y Sasabuchi Y Takazawa T , et al. Long-term prognosis following early rehabilitation in the ICU: a retrospective cohort study. Crit Care Med 2023; 51: 1054–1063. 36988323 10.1097/CCM.0000000000005862 PMC10335737

[11] Morris PE Goad A Thompson C , et al. Early intensive care unit mobility therapy in the treatment of acute respiratory failure. Crit Care Med 2008; 36: 2238–2243. 18596631 10.1097/CCM.0b013e318180b90e

[12] van Willigen Z Collings N Richardson D , et al. Quality improvement: the delivery of true early mobilisation in an intensive care unit. BMJ Qual Improv Rep 2016; 5: u211734. 10.1136/bmjquality.u211734.w4726 PMC5223689 28090326

[13] Cusack R Bates A Mitchell K , et al. Improving physical function of patients following intensive care unit admission (EMPRESS): protocol of a randomised controlled feasibility trial. BMJ Open 2022; 12: e055285. 10.1136/bmjopen-2021-055285 PMC9014051 35428629

[14] Herridge MS Chu LM Matte A , et al. The RECOVER program: disability. Risk groups and 1-year outcome after 7 or more days of mechanical ventilation. Am J Respir Crit Care Med 2016; 194: 831–844. 26974173 10.1164/rccm.201512-2343OC

[15] Sessler CN Gosnell MS Grap MJ , et al. The Richmond agitation–sedation scale. Am J Respir Crit Care Med 2002; 166: 1338–1344. 12421743 10.1164/rccm.2107138

[16] Ely EW Truman B Shintani A , et al. Monitoring sedation status over time in ICU patients: reliability and validity of the Richmond agitation-Sedation Scale (RASS). JAMA 2003; 289: 2983–2991. 12799407 10.1001/jama.289.22.2983

[17] Moss M Nordon-Craft A Malone D , et al. A randomized trial of an intensive physical therapy program for patients with acute respiratory failure. Am J Respir Crit Care Med 2016; 193: 1101–1110. 26651376 10.1164/rccm.201505-1039OC PMC4872662

[bibr18-17511437251328899] Denehy L Skinner EH Edbrooke L , et al. Exercise rehabilitation for patients with critical illness: a randomized controlled trial with 12 months of follow-up. Crit Care 2013; 17: R156. 10.1186/cc12835 PMC4056792 23883525

[19] Kho ME Molloy AJ Clarke FJ , et al. Multicentre pilot randomised clinical trial of early in-bed cycle ergometry with ventilated patients. BMJ Open Respir Res 2019; 6: e000383. 10.1136/bmjresp-2018-000383 PMC6424272 30956804

[20] Wright SE Thomas K Watson G , et al. Intensive versus standard physical rehabilitation therapy in the critically ill (EPICC): a multicentre, parallel-group, randomised controlled trial. Thorax 2018; 73: 213–221. 28780504 10.1136/thoraxjnl-2016-209858 PMC5870467

[21] Kho ME Molloy AJ Clarke FJ , et al. Multicentre pilot randomised clinical trial of early in-bed cycle ergometry with ventilated patients. BMJ Open Resp Res 2019; 6: e000383. 10.1136/bmjresp-2018-000383 PMC6424272 30956804

[22] Puthucheary ZA Denehy L. Exercise Interventions in critical illness survivors: understanding inclusion and stratification criteria. Am J Respir Crit Care Med 2015; 191: 1464–1467. 26075426 10.1164/rccm.201410-1907LE

[23] Fuest KE Ulm B Daum N , et al. Clustering of critically ill patients using an individualized learning approach enables dose optimization of mobilization in the ICU. Crit Care 2023; 27: 1. 36597110 10.1186/s13054-022-04291-8 PMC9808956

[bibr24-17511437251328899] Winkelman C Sattar A Momotaz H , et al. Dose of early therapeutic mobility: does frequency or intensity matter? Biol Res Nurs 2018; 20: 522–530. 29902939 10.1177/1099800418780492 PMC6346319

[bibr25-17511437251328899] Paton M Lane R Paul E , et al. Mobilization during critical illness: a higher level of mobilization improves health status at 6 months, a secondary analysis of a prospective cohort study. Crit Care Med 2021; 49: E860–E869. 10.1097/CCM.0000000000005058 33967203

[bibr26-17511437251328899] Lord RK Mayhew CR Korupolu R , et al. ICU early physical rehabilitation programs: financial modeling of cost savings. Crit Care Med 2013; 41: 717–724. 23318489 10.1097/CCM.0b013e3182711de2

[27] Langhorne P Wu O Rodgers H , et al. A very early rehabilitation trial after stroke (AVERT): a phase III, multicentre, randomised controlled trial. Health Technol Assess 2017; 21: 1–120. 10.3310/hta21540 PMC5641820 28967376

[28] Ruo Yu L Jia Jia W Meng Tian W , et al. Optimal timing for early mobilization initiatives in intensive care unit patients: a systematic review and network meta-analysis. Intensive Crit Care Nurs 2024; 82: 103607. 38158250 10.1016/j.iccn.2023.103607

[29] Covinsky KE Pierluissi E Johnston CB. Hospitalization-associated disability: “she was probably able to ambulate, but i’m not sure”. JAMA 2011; 306: 1782–1793. 22028354 10.1001/jama.2011.1556

[bibr30-17511437251328899] Puthucheary ZA Rawal J McPhail M , et al. Acute skeletal muscle wasting in critical illness. JAMA 2013; 310: 1591–1600. 24108501 10.1001/jama.2013.278481

[31] Levine S Nguyen T Taylor N , et al. Rapid disuse atrophy of diaphragm fibers in mechanically ventilated humans. N Engl J Med 2008; 358: 1327–1335. 18367735 10.1056/NEJMoa070447

[32] Norrenberg M Vincent JL. A profile of European intensive care unit physiotherapists. European Society of Intensive Care Medicine. Intensive Care Med 2000; 26: 988–994. 10990117 10.1007/s001340051292

[bibr33-17511437251328899] Hodgson C Bellomo R Berney S , et al. Early mobilization and recovery in mechanically ventilated patients in the ICU: a bi-national, multi-centre, prospective cohort study. Crit Care 2015; 19: 81. 25715872 10.1186/s13054-015-0765-4 PMC4342087

[34] Hodgin KE Nordon-Craft A McFann KK , et al. Physical therapy utilization in intensive care units: results from a national survey. Crit Care Med 2009; 37: 561–566. 19114903 10.1097/CCM.0b013e3181957449 PMC2908523

[35] Needham DM Korupolu R Zanni JM , et al. Early physical medicine and rehabilitation for patients with acute respiratory failure: a quality improvement project. Arch Phys Med Rehabil 2010; 91: 536–542. 20382284 10.1016/j.apmr.2010.01.002

[bibr36-17511437251328899] Harris CL Shahid S. Physical therapy-driven quality improvement to promote early mobility in the intensive care unit. Proc 2014; 27: 203–207. 10.1080/08998280.2014.11929108 PMC4059563 24982559

[bibr37-17511437251328899] Kho ME Molloy AJ Clarke FJ , et al. TryCYCLE: a prospective study of the safety and feasibility of early in-bed cycling in mechanically ventilated patients. PLoS One 2016; 11: e0167561. 10.1371/journal.pone.0167561 PMC5193383 28030555

[38] Hodgson CL Bailey M Bellomo R , et al. A binational multicenter pilot feasibility randomized controlled trial of early goal-directed mobilization in the ICU. Crit Care Med 2016; 44: 1145–1152. 26968024 10.1097/CCM.0000000000001643

[39] Lorenz M Fuest K Ulm B , et al. The optimal dose of mobilisation therapy in the ICU: a prospective cohort study. J Intensive Care 2023; 11: 56. 37986100 10.1186/s40560-023-00703-1 PMC10658796

[40] Connolly BA Barclay M Davies C , et al. PRACTICE: development of a core outcome set for trials of physical rehabilitation in critical illness. Ann Am Thorac Soc 2024; 21: 1742–1750. 39189977 10.1513/AnnalsATS.202406-581OC PMC11622824

[41] Duceau B Blatzer M Bardon J , et al. Using a multiomics approach to unravel a septic shock specific signature in skeletal muscle. Sci Rep 2022; 12: 18776–18776. 36335235 10.1038/s41598-022-23544-8 PMC9637214

[42] Supinski GS Schroder EA Callahan LA. Mitochondria and critical illness. Chest 2020; 157: 310–322. 31494084 10.1016/j.chest.2019.08.2182 PMC7005375

[43] Flockhart M Nilsson LC Tais S , et al. Excessive exercise training causes mitochondrial functional impairment and decreases glucose tolerance in healthy volunteers. Cell Metab 2021; 33: 957–970.e956. 10.1016/j.cmet.2021.02.017 33740420

