# Supplementary material for: Improving physical function with physiotherapy assistants following intensive care unit admission (EMPRESS): A randomised controlled feasibility study
Source: J Intensive Care Soc. 2025 May 16;26(3):317–25. doi: 10.1177/17511437251328899 (PMC12084218; doi:10.1177/17511437251328899)
Supplement: sj-docx-1-inc-10.1177_17511437251328899 – Supplemental material for Improving physical function with physiotherapy assistants following intensive care unit admission (EMPRESS): A randomised controlled feasibility study [file sj-docx-1-inc-10.1177_17511437251328899.docx]

**Supplementary information**

| **Supplementary Table 1a. Sites staff structure and patient accruals** | | | |
| --- | --- | --- | --- |
|  |  |  |  |
|  | **Site 1** | **Site 2** | **Site 3** |
| Hospital | District | Teaching | District |
| Beds | ~12 | 35 | 13 |
| Physiotherapy Staff (WTE)   1. Senior 2. Middle 3. Lower 4. Assistants | 1  1  1  1.6* | 0.5  1  1  1** | 0.5  2  1  0 |
| Patient accrual   1. Total 2. Patients/month | 12  0.6 | 27  1.2 | 7  0.8 |

WTE = whole time equivalent; * In post, 1 WTE reallocated to study; **Employed solely for the study intervention.

| **Supplementary Table 1b Documented Usual care interventions by site** | | | |
| --- | --- | --- | --- |
|  |  |  |  |
| Site 1 | Daily physiotherapy assessments and respiratory care were provided to all patients. Limited passive and active movements were performed while patients were in bed. For longer-term patients, a higher level of in-bed mobilisation was introduced after 10 days. However, no documentation of out-of-bed mobilisation was found for any patients while they were | | |
|  |  |  |  |
| Site 2 | No routine daily physiotherapy interventions were provided. Interventions were given as clinically indicated to manage respiratory secretions. Passive range of motion exercises were performed intermittently. One patient was mobilised to the edge of the bed while intubated by day 7. Otherwise, limited active mobilisation was documented. No documentation of out-of-bed mobilisation was found for any patients while they were | | |
|  |  |  |  |
| Site 3 | Daily physiotherapy assessments, respiratory care, and passive range of motion exercises were provided from admission. Progress to sitting on the edge of the bed with the endotracheal tube common by day 7. No documentation of out-of-bed mobilisation was found for any patients while they were intubated | | |

| **Supplementary Table 2. Adverse events and safety criteria for delivery and ceasing interventions** | | |
| --- | --- | --- |
|  |  |  |
|  | **Criteria to commence physiotherapy** | **Criteria to stop / withhold physiotherapy intervention** |
| Blood pressure | MAP 60 – 100 mmHg, no change in vasopressor dose requirement for preceding 2 hours | Catecholamine resistant hypotension with MAP < 60 mmHg |
| Heart rate | Between 40-140 bpm | <40 or >140 bpm |
| Respiratory rate | Sustained < 40 breaths/min |  |
| Temperature |  | >40 °C |
| Oxygen requirement | If FiO2 >0.8 for passive exercise only |  |
|  | FiO2 <0.8 and PEEP<15 cmH_2_O |  |
| Desaturation |  | Sats fall <85% for > 1 minute |
| Adverse events |  | - Fall |
|  |  | - Unplanned extubation |
|  |  | - Acute bleeding |
|  |  | - New onset arrhythmia |
|  |  | - Signs/symptoms of acute myocardial ischaemia |
|  |  | - Patient pain/distress - Clinical team decide therapy intervention not appropriate - Refusal by patient or representative |

| **Supplementary Table 3. Secondary clinical outcome assessments** | | | | | | | | |
| --- | --- | --- | --- | --- | --- | --- | --- | --- |
|  | | | | | | | | |
|  | Day 1 | Day 3 | Day 7 | Awakening | Weekly | ICU discharge | Hospital discharge | 3 months post discharge |
| **Muscle assessment**   - MRCss - HHD |  |  |  | X  X | X  X | X  X | X  X |  |
| **Physical function**   - CPAX - ICU Mobility - PFITs - TUG - Clinical Frailty - Barthel - 6MWT | X | X | X | X  X | X  X  X | X  X  X  X  X  X  X | X  X  X  X | X  X  X  X |
| **HRQL**   - WHODAS2 - HADS - EQ5D-5L - IES-r |  |  |  |  |  |  |  | X  X  X  X |

Physical Function ICU Test–scored (PFIT-s) ^1, 2^; Medical Research Council Manual Muscle Test Sum Score (MRC-ss) ^3, 4^; handheld dynamometry( HDD) ^5^; Chelsea Critical Care Physical Assessment Tool (CPAx) ^6^; ICU Mobility Scale ^7^; Timed Up and Go (TUG) ^8, 9^ Clinical Frailty Score (CFS) ^10-12^Six-minute walk tests (6MWT) ^13^. Preadmission Barthel Index and CFS were to be assessed by proxy on admission from a family member or next of kin. Health-related quality of life (HRQoL), WHODAS 2.0,^14^ Hospital Anxiety and Depression Scale score (HADS) ^15, 16^; Impact of Event(IES-r) ^17-19^, Euroqol- 5 Dimension- 5 Level (EQ- 5D- 5L) ^20^

| **Supplementary Table 4. Patient Outcomes at ICU discharge (secondary)** | | | | |  |
| --- | --- | --- | --- | --- | --- |
|  |  | **Usual care**  **(N=13)** | **Usual care + intervention**  **(N=14)** | **Total**  **(N=27)*** | |
|  |  |  |  |  | |
| **PFIT-s median (IQR)** |  | 3.9 (0 – 6.4) | 6.8 (3.9 – 7.9) | 4.4 (2 – 7.1) | |
|  |  |  |  |  | |
| **ICU mobility median(IQR)**  **(ordinal score scale 0-10)** |  | 4 (3 – 8) | 7.5 (4 – 10) | 7 (3 – 9) | |
| **TUG median (IQR)**** |  | **60 (11 – 67)**  **N=3** | **18 (13 – 24.5)**  **N=8** | **21 (13 – 30)**  **N=11** | |
|  |  |  |  |  | |
| **Frailty**  **(score 1 – 9)** |  | 6 (3 – 6) | 6 (3 – 7) | 6 (3 – 6) | |
| **Barthel median (IQR)** |  | 40 (15 – 45) | 32.5 (5 – 85) | 35 (5 – 6.5) | |
| **CPAx median (IQR)** |  | 28 (17 – 38) | 34.5 (16 – 44) | 32 (16 – 43) | |
| **MRCSS median (IQR)** |  | 36 (30 – 48) | 45.5 (35 – 58) | 43 (30 – 54) | |
| **HHD*** median (IQR)** |  | **LH 9 (6.7-25.4)**  **RH 15 (5-32)**  **N=7** | **LH 15.5(10-21.8)**  **RH 16.4(8-29.2)**  **N=10** | **LH 12 (8-21.8)**  **RH15.9 (8-9.2)**  **N=17** | |

*Surviving patients with available data at ICU discharge.

** Only 11 patients completed the TUG test: 3 and 8 patients in the usual care group and intervention groups respectively.

*** Only 17 patients completed HHD: 7 and 10 patients in the usual care group and intervention groups respectively. LH = Left Hand, RH = Right Hand.

NB Of the proposed assessments of physical function, two of the three sites were unable to complete 6-minute walk test according to ATS guidelines (due to limited space and staff to be able perform the test) and therefore this outcome is not presented.

**References**

1. Denehy L, de Morton NA, Skinner EH, et al. A physical function test for use in the intensive care unit: validity, responsiveness, and predictive utility of the physical function ICU test (scored). *Phys Ther* 2013; 93: 1636-1645. 2013/07/28. DOI: 10.2522/ptj.20120310.

2. Skinner EH, Berney S, Warrillow S, et al. Development of a physical function outcome measure (PFIT) and a pilot exercise training protocol for use in intensive care. *Crit Care Resusc* 2009; 11: 110-115. 2009/06/03.

3. Fan E, Ciesla ND, Truong AD, et al. Inter-rater reliability of manual muscle strength testing in ICU survivors and simulated patients. *Intensive Care Med* 2010; 36: 1038-1043. 2010/03/10. DOI: 10.1007/s00134-010-1796-6.

4. Hermans G, Clerckx B, Vanhullebusch T, et al. Interobserver agreement of Medical Research Council sum-score and handgrip strength in the intensive care unit. *Muscle Nerve* 2012; 45: 18-25. 2011/12/23. DOI: 10.1002/mus.22219.

5. Vanpee G, Segers J, Van Mechelen H, et al. The interobserver agreement of handheld dynamometry for muscle strength assessment in critically ill patients. *Crit Care Med* 2011; 39: 1929-1934. 2011/05/17. DOI: 10.1097/CCM.0b013e31821f050b.

6. Corner EJ, Wood H, Englebretsen C, et al. The Chelsea critical care physical assessment tool (CPAx): validation of an innovative new tool to measure physical morbidity in the general adult critical care population; an observational proof-of-concept pilot study. *Physiotherapy* 2013; 99: 33-41. 2012/12/12. DOI: 10.1016/j.physio.2012.01.003.

7. Hodgson C, Needham D, Haines K, et al. Feasibility and inter-rater reliability of the ICU Mobility Scale. *Heart Lung* 2014; 43: 19-24. 2014/01/01. DOI: 10.1016/j.hrtlng.2013.11.003.

8. Bischoff HA, Stähelin HB, Monsch AU, et al. Identifying a cut-off point for normal mobility: a comparison of the timed 'up and go' test in community-dwelling and institutionalised elderly women. *Age Ageing* 2003; 32: 315-320. 2003/05/02. DOI: 10.1093/ageing/32.3.315.

9. Ng SS and Hui-Chan CW. The timed up & go test: its reliability and association with lower-limb impairments and locomotor capacities in people with chronic stroke. *Arch Phys Med Rehabil* 2005; 86: 1641-1647. 2005/08/09. DOI: 10.1016/j.apmr.2005.01.011.

10. Bagshaw M, Majumdar SR, Rolfson DB, et al. A prospective multicenter cohort study of frailty in younger critically ill patients. *Crit Care* 2016; 20: 175. 2016/06/07. DOI: 10.1186/s13054-016-1338-x.

11. Bagshaw SM, Stelfox HT, Johnson JA, et al. Long-term association between frailty and health-related quality of life among survivors of critical illness: a prospective multicenter cohort study. *Crit Care Med* 2015; 43: 973-982. 2015/02/11. DOI: 10.1097/ccm.0000000000000860.

12. Rockwood K, Song X, MacKnight C, et al. A global clinical measure of fitness and frailty in elderly people. *Cmaj* 2005; 173: 489-495. 2005/09/01. DOI: 10.1503/cmaj.050051.

13. ATS statement: guidelines for the six-minute walk test. *Am J Respir Crit Care Med* 2002; 166: 111-117. 2002/07/02. DOI: 10.1164/ajrccm.166.1.at1102.

14. Üstün TB and Organisation WH. *Measuring health and disability: Manual for WHO Disability Assessment Schedule WHODAS 2.0.* Geneva: World Health Organisation, 2010.

15. Bjelland I, Dahl AA, Haug TT, et al. The validity of the Hospital Anxiety and Depression Scale. An updated literature review. *J Psychosom Res* 2002; 52: 69-77. 2002/02/08. DOI: 10.1016/s0022-3999(01)00296-3.

16. Zigmond AS and Snaith RP. The Hospital Anxiety and Depression Scale. *Acta Psychiat Scand* 1983; 67: 361-370. DOI: DOI 10.1111/j.1600-0447.1983.tb09716.x.

17. Schwarzwald J, Solomon Z, Weisenberg M, et al. Validation of the Impact of Event Scale for psychological sequelae of combat. *J Consult Clin Psychol* 1987; 55: 251-256. 1987/04/01. DOI: 10.1037//0022-006x.55.2.251.

18. Weiss DS, & Marmar, C. R. The Impact of Event Scale - Revised. In: J. Wilson TMK (ed) *Assessing psychological trauma and PTSD*. New York: Guilford, 1996, pp.399-411.

19. Horowitz M, Wilner N and Alvarez W. Impact of Event Scale: a measure of subjective stress. *Psychosom Med* 1979; 41: 209-218. DOI: 10.1097/00006842-197905000-00004.

20. Herdman M, Gudex C, Lloyd A, et al. Development and preliminary testing of the new five-level version of EQ-5D (EQ-5D-5L). *Qual Life Res* 2011; 20: 1727-1736. 2011/04/12. DOI: 10.1007/s11136-011-9903-x.
